# RNAi-Mediated Knock-Down of *transformer* and *transformer 2* to Generate Male-Only Progeny in the Oriental Fruit Fly, *Bactrocera dorsalis* (Hendel)

**DOI:** 10.1371/journal.pone.0128892

**Published:** 2015-06-09

**Authors:** Guiqing Liu, Qiang Wu, Jianwei Li, Guifen Zhang, Fanghao Wan

**Affiliations:** 1 Department of Biological Invasions, State Key Laboratory for Biology of Plant Diseases and Insect Pests, Institute of Plant Protection, Chinese Academy of Agricultural Sciences, Beijing, China; 2 Guangdong Key Laboratory of Integrated Pest Management in Agriculture, Guangdong Public Laboratory of Wild Animal Conservation and Utilization, Guangdong Entomological Institute, Guangzhou, China; 3 Agricultural Genome Institute at Shenzhen, Chinese Academy of Agricultural Sciences, Shenzhen, China; International Atomic Energy Agency, AUSTRIA

## Abstract

The *transformer* (*tra*) gene appears to act as the genetic switch that promotes female development by interaction with the *transformer2* (*tra-2*) gene in several dipteran species including the Medfly, housefly and *Drosophila melanogaster*. In this study, we describe the isolation, expression and function of *tra* and *tra-2* in the economically important agricultural pest, the oriental fruit fly, *Bactrocera dorsalis* (Hendel). *Bdtra* and *Bdtra-2* are similar to their homologs from other tephritid species. *Bdtra* demonstrated sex-specific transcripts: one transcript in females and two transcripts in males. In contrast, *Bdtra-2* only had one transcript that was common to males and females, which was transcribed continuously in different adult tissues and developmental stages. *Bdtra-2* and the female form of *Bdtra* were maternally inherited in eggs, whereas the male form of *Bdtra* was not detectable until embryos of 1 and 2 h after egg laying. Function analyses of *Bdtra* and *Bdtra-2* indicated that both were indispensable for female development, as nearly 100% males were obtained with embryonic RNAi against either *Bdtra* or *Bdtra-2*. The fertility of these RNAi-generated males was subsequently tested. More than 80% of RNAi-generated males could mate and the mated females could lay eggs, but only 40-48.6% males gave rise to progeny. In XX-reversed males and intersex individuals, no clear female gonadal morphology was observed after dissection. These results shed light on the development of a genetic sexing system with male-only release for this agricultural pest.

## Introduction

Sexual reproduction is an essential and universal phenomenon among animals to maintain species numbers and diversity. In insects, multiple genetic mechanisms have been identified for sex determination [1–5]. The oriental fruit fly, *Bactrocera dorsalis* (Hendel) is classified in the *B*. *dorsalis* complex, which is a group of tropical fruit flies contains 75 morphologically similar taxa [6]. Recently, *B*. *dorsalis* was demonstrated to be the senior synonym of *B*. *papaya* Drew and Hancock and *B*. *invaden* Drew, Tsuruta and White, but not *B*. *carambolae* [7]. *B*. *dorsalis* is one of the most destructive pests throughout Southeast Asia and a number of the Pacific Islands and is considered to be one of the most important pest species in world agriculture by some entomologists [6–7]. The distribution of *B*. *dorsalis* includes much of the tropical and subtropical regions and extends into warm temperate areas such as southern Mediterranean Europe [8]. As a result of its widespread distribution, invasive ability and potential impact on market access, *B*. *dorsalis* is considered to be a major threat to many countries fruit industries. In order to control the population of this species and other economically important agricultural pests, an effective, biologically-based control system has been developed, termed the sterile insect technique (SIT) [9]. For most SIT targets, large scale sex separation is considered highly desirable or essential for the release of only males [10–11]. Thus, a comprehensive understanding of sexual differentiation in the *B*. *dorsalis* species offers an opportunity to promote the development of novel sexing strategies and genetic control techniques to fight this pest.

Although the primary signal and subordinate genes for sex determination in *B*. *dorsalis* are currently unknown, the genetic cascade regulating sexual development has been well characterized in *Drosophila* [12]. Sex determination starts with the primary signal, which then regulates a cascade of genes and ends with transcription of the conserved *doublesex* (*dsx*) gene that controls sexual differentiation. The diverse mechanisms for sex determination mainly result from the presence of complicated primary signals. In *Drosophila melanogaster*, the primary signal is the ratio between X chromosomes and autosomal chromosomes. When the ratio is 1.0 (XX: AA), the sex determination master gene *Sex lethal* (*sxl*) is active; while the ratio is 0.5 (X: AA), *sxl* remains inactive. A previous study suggests that the primary signal is the X-linked signal elements (XSEs) which function to activate the *sxl* gene in females [13]. As a genetic switch, *sxl* is only turned on in females and promotes female somatic sexual development via its auto-regulation [14]. The protein made by the *sxl* transcript directs female-specific splicing of the downstream gene *tra*, resulting in the production of functional Tra protein only in females [15]. The Tra protein, together with the product of the gene *tra-2*, activates female-specific splicing of *doublesex* (*dsx*), generating one female-specific Dsx protein [16]. In contrast, when *sxl* is inactive, a non-functional, truncated Tra peptide is produced, resulting from the male-specific splicing of *tra*. Thus, when the interaction between Tra and Tra-2 is absent, one male-specific Dsx protein is generated from the default splicing of *dsx* pre-mRNA. Both male and female specific Dsx proteins are functional but control sexual dimorphism in opposite ways [14, 16].

In tephritid fruit flies, the primary signal that controls this pathway is the *Dominant Male Determiner* (*M*). The *sxl* ortholog in tephritids is not sex-specifically expressed and appears to play no role in sex determination. Although *M* hasn’t been characterized, *tra* and *tra-2* have been identified in several species, including *Ceratitis capitata* [17–18], *Musca domestica* [19–20], *Bactrocera oleae* [21], *Lucilia cuprina* [22] and *Anastrepha* species [23–25]. In these species, transient, embryonic knock-down of *tra* led to XX males, suggesting that *tra* initiates an auto-regulatory mechanism in early embryos, in contrast to *Drosophila*. In addition, evidence that *tra-2* is required to establish and maintain female somatic sexual development was first reported in *M*. *domestica* [19], then in *C*. *capitata* [18] and *A*. *suspensa* [25], but not in *Bactrocera* species. In these species, injection of *tra-2* dsRNA in XX female embryos led to phenotypic male development. As released sterile females do not contribute to sterility in the field population and can cause losses by female stinging or female transmitting disease, a several-fold advantage has been found in male-only release program [10–11]. The ability to reverse XX females to phenotypic males is highly advantageous to biological control programs such as SIT programs that base on male-only release, as well as the development of transgenic sexing strategies based on female-specific lethality [26–28] and transgenic autocidal strategies where released males transfer conditional lethal genes to their progeny [29–30]. In medfly, transgenic RNAi *transformer* has already proven to be effective in heat shock induced production of male-only progeny. It is considered to be a promising strategy for the development of novel sexing strains [31].

In *B*. *dorsalis*, sequence analysis of the *dsx* homolog reveals the presence of four putative Tra/Tra-2 binding sites located at female-specific exon 4 [32]. The four conserved 13 nucleotide repeat elements suggest that the Dsx protein in *B*. *dorsalis* retains the conserved essential role as a double switch regulator in sexual development. In order to obtain a comprehensive understanding of the roles of *tra* and *tra-2* in sex determination and fertility in *B*. *dorsalis*, the genomic and cDNA structure of *Bdtra* and *Bdtra-2* genes were isolated and analyzed. The sex-specific splicing of *Bdtra* was assessed using RT-PCR. Embryonic RNAi was employed to determine the effects of *Bdtra* and *Bdtra-2* on sex determination and fertility. The results of this study advance our capability to generate male-only progeny with the ability to mate by a molecular approach, leading to an important advance toward improving SIT in *B*. *dorsalis*.

## Methods and Materials

### Rearing of *B*. *dorsalis*

The oriental fruit flies used in this work were generated in an inbred wild-type colony. Flies were reared at 27°C with 75–80% relative humidity, under a cycle of 14/10 h light/dark, and fed an artificial diet consisting of 1g cane sugar and 8g yeast extract. Newly laid eggs were collected and transferred onto the surface of larval medium consisting of 2.5 g agar, 4 g sodium benzoate, 2.86 g nipagin, 4 g tissues, 35 g brewer’s yeast, 95 g corn meal and 4 ml concentrated HCl in a 1 liter diet. Larvae burrowed into the food and emerged again when ready to pupate. Before pupation, they were transferred into small plastic boxes with sand. Pupae were kept at 27°C until adults emerged.

### Isolation of *Bdtra* and *Bdtra-2*

To isolate *Bdtra* and *Bdtra-2*, total RNA was extracted from male and female adults using TRIzol Reagent (Invitrogen). For each sex, three cDNA pools were generated: 1) the first strand of cDNA was synthesized using 2 μg total RNA, oligo-dT primer and Quant reverse transcriptase based on instructions from the manual of the FastQuant RT Kit (with gDNase, TianGen); 2) 5’-RACE cDNA; and 3) 3’-RACE cDNA using the BD SMARTer RACE cDNA Amplification Kit (BD Biosciences). First, *Bdtra* and *Bdtra-2* cDNAs were isolated using RT-PCRs on adult male and female cDNA pool 1 template with primers 260+/1329- and AF2/AR1 at an annealing temperature of 50°C and 60°C, respectively. These primers were designed on the base of cDNA sequences from *B*. *oleae* by aligning Tra and Tra-2 protein sequences from *C*. *capitata* (GenBank: AF434936; EU999754), *B*. *oleae* (GenBank: AJ715413; AJ547623), and *A*. *suspensa* (GenBank: JN597286; JN597290), respectively (S1 and S2 Figs). Second, RACE PCRs were performed on cDNA pool 2 and 3 to obtain 5’UTR and 3’UTR for each sex, following the manual instructions. Primers 286+/1043+ and 514-/286- were used to isolate 3’UTR and 5’UTR of *Bdtra* by nested PCR, respectively. For *Bdtra-2*, primers 354+ and 228- were used. Third, genomic DNA was extracted using TIANamp Genomic DNA Kit (TianGen). Introns were amplified using PCRs on genomic DNA with primers 3+/299-, 225+/706- and 655+/1285- at 60°C for annealing. For *Bdtra-2*, primers 9+/366- and 358+/1185- were used with an annealing temperature of 60°C. All the PCR products were purified and cloned into T vector following the manual of the pEASY-T1 Cloning Kit (TransGen). The positive recombinants were cultured and sequenced subsequently.

### Sequence analyses

Sequence alignment and similarity analyses were performed using the NCBI website. Multiple sequence alignments of Tra and Tra-2 protein sequence were analyzed with the online ClustalW2 software (http://www.ebi.ac.uk/Tools/msa/clustalw2/). Phylogenic trees of Tra and Tra-2 were reconstructed using the neighbor-joining tree-building method. The reliability of the resulting topologies was tested by the bootstrap method. Phylogenetic trees were rooted using the *tra* or *tra-2* gene of Hymenoptera. Exons and introns of *Bdtra-2* were identified by aligning sequences using the Spidey program (http://www.ncbi.nlm.nih.gov/spidey/).

### Expression patterns of *Bdtra* and *Bdtra-2*

To analyze the expression patterns of *Bdtra* and *Bdtra-2*, cDNA templates were prepared from total RNA extracted from different developmental stages and tissues. The specimens of eggs, larvae and pupae were collected from mixed sexes and carefully staged. Tissues from adults were dissected in DEPC-treated PBS buffer. All the specimens were homogenized immediately in liquid nitrogen. The total RNAs were extracted using TRIzol Reagent (Invitrogen) and 2 μg qualified total RNA of each speciesmen was used to synthesized the first trand cDNA following the manual of the FastQuant RT Kit (with gDNase, TianGen). RT-PCR was performed to analyze the sex-specific patterns of *Bdtra* using primers 225+/706- and ms43+/706- both at an annealing temperature of 60°C. For *Bdtra-2*, primers 9+/1185- were used at a 60°C annealing temperature. RT-PCR product of the reference gene *a-tubulin* (*a-tub*) using primers *a-tub* +/ *a-tub*—was used as a control.

### RNAi knock-down of *Bdtra* and *Bdtra-2*

For double-stranded RNA synthesis, two fragments of *Bdtra* were amplified with primers 79+/576- and 613+/1205- from female cDNA and one *Bdtra-2* fragment with primers 422+/732-. An *EGFP* control fragment was isolated with primers 27+/688- on the vector 1201 containing *EGFP* [33]. A T7 binding site for the T7 RNA polymerase was introduced into these primers. DsRNAs were generated using the Ambion Megascript T7 Kit and then purified with Ambion MEGAclear following the manual instructions. Function analyses were performed by microinjection of dsRNA into embryos as described by Handler and James [34]. Embryos were collected half an hour after the eggs were laid, dechorionated and microinjected with 2 mg/ml dsRNA solution. Approximately 400–500 embryos were injected for each treatment.

### Fertility test

Adults that developed from eggs injected with *EGFP*, *Bdtra* and *Bdtra-2* dsRNAs were counted and examined for their sexual morphological traits. Males were reared and used to test fertility under standard laboratory condition. For each RNAi treatment, 35 cages containing all intersex individuals were set up on day 6 after the adults emerged. One RNAi male was backcrossed to two virgin wild-type females in each cage. Mating was observed every 30 min during the dark photoperiod, and eggs from each cage were collected continuously for 5 days. The newly hatched larvae were then transferred onto host fruit until the adults emerged. After the fertility test, the RNAi males from each cage were dissected in DEPC-treated 1×PBS buffer for analysis of their internal genital structures. The residual bodies without gonads of all the XX-reversed males, intersex individuals, and a portion of the XY males without gonads were collected and homogenized immediately in liquid nitrogen for total RNA extraction. The first strand cDNA was synthesized using 2 μg of total RNA and reverse transcribed using the FastQuant RT Kit (TianGen). RT-PCR was performed to analyze the splicing pattern of *tra* and *dsx* from RNAi males. The specific primers 225+/706- were used to amplify *Bdtra*. To amplify *Bddsx*, non-sex-specific primer 1074+, female-specific primer f1072- and male-specific primer m2015- were mixed to use. Both RT-PCRs were at a 60°C annealing temperature.

All the primers used in this study were listed in S1 Table.

### Imaging

The images of the flies of each RNAi phenotype were taken using a Keyence VHX-2000 Microscope.

## Results

### Isolation of *Bdtra* and *Bdtra-2*

**Fig 1:**
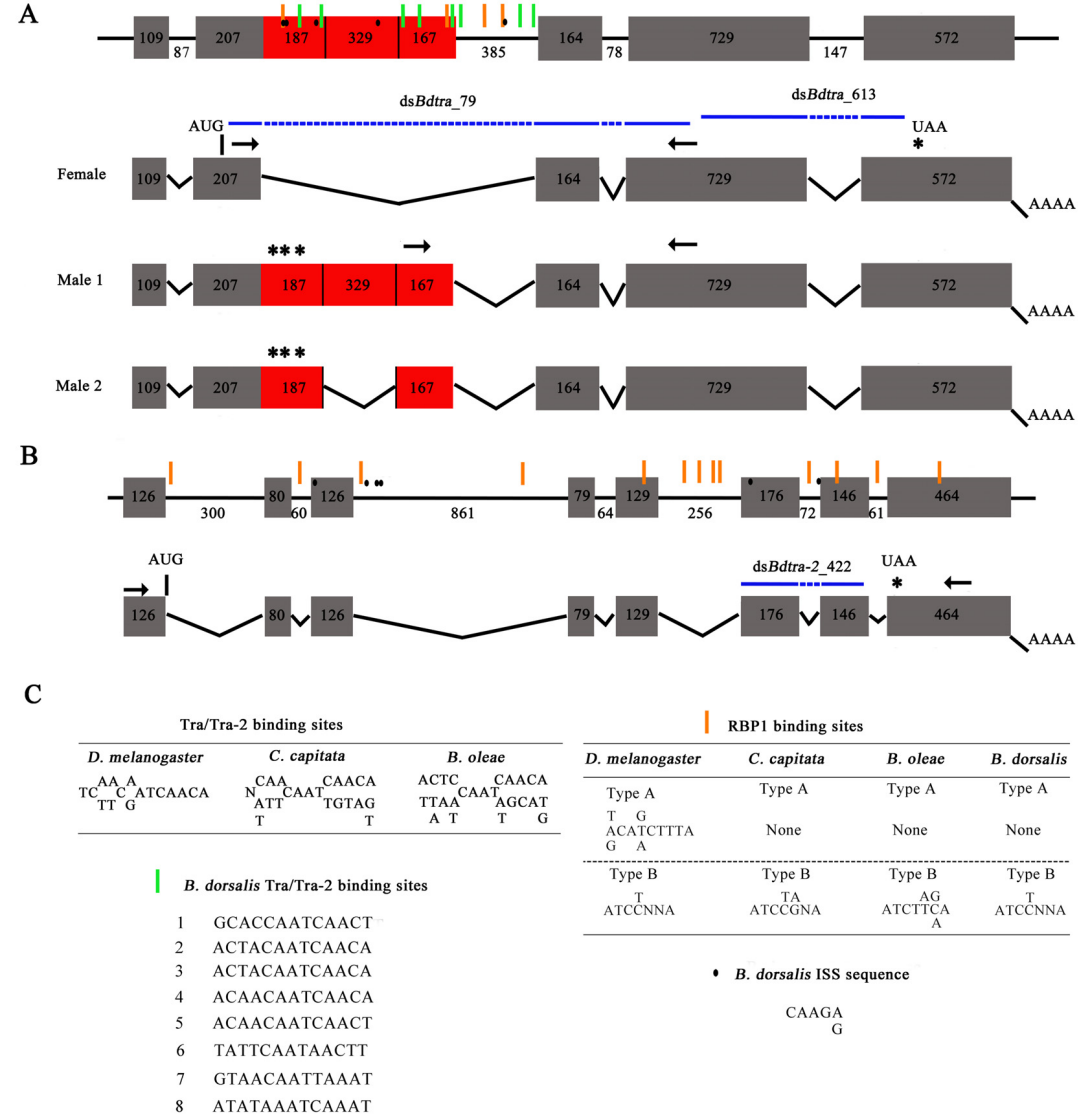
Genomic and cDNA structure of *Bdtra* and *Bdtra-2*. (A) Genomic structure of *Bdtra* showing exons (boxes), introns (lines), sex-specific transcripts in males and females and the locations of the Tra/Tra-2, RBP1 and ISS binding sites. (B) Genomic structure of *Bdtra-2* showing exons (boxes), introns (lines) and the non-sex-specific transcript. (C) The consensus sequences of Tra/Tra-2, RBP1 and ISS binding sites. Horizontal arrows indicate the positions of primer pair 225+/706- and ms43+/706- (A) and primer pair 9+/1185- (B) for the expression analyses and numbers down the lines and inside the boxes represent the length of introns and exons, respectively. Vertical bars represent the position of translation start codons. Red boxes stand for the male-specific exons with the translation stop codons marked by asterisks above. Blue horizontal lines indicate the positions of the double strand RNAs named ds*Bdtra*_79, ds*Bdtra*_613 (A) and ds*Bdtra-2*_422 (B) for RNAi.

*Bdtra* was isolated in three steps. First, PCRs were performed on adult male and female cDNA using primers designed on the basis of the conserved amino acid motifs in the *tra* homologs of *B*. *oleae*, *C*. *capitata* and *A*. *suspensa*. Second, 5’- and 3’- RACE PCRs were performed on RACE cDNA pools to obtain full-length cDNA sequences. Third, introns were identified by PCRs on genomic DNA using exon-specific primers. One female-specific transcript (1729 bp containing an open reading frame of 1269 bp) (GenBank: KP342058) and two male-specific transcripts (2083 bp and 2412 bp) (GenBank: KP342060 and KP342059) were found. These transcripts were identified as homologs of *tra* gene based on similarities to other species including *B*. *oleae*, *A*. *obliqua* and *C*. *capitata* using BlastX alignment. Due to the sex-specific splicing of *Bdtra* pre-mRNA, female transcript produces fully functional Tra protein of 422 amino acids, whereas male transcripts produce non-functional truncated Tra peptides due to the presence of several in-frame stop codons. The presence of Tra/Tra-2 binding sites, intronic splicing silencer sequence (ISS) and RBP1 binding sites has been reported in several insect species [17, 23, 25]. On the basis of sequence similarity, we searched for these sites in the differentially spliced intron of *Bdtra*. Eight Tra/Tra-2 binding sites, four RBP1 binding sites and five ISS sites were found (Fig 1A and 1C; S3 Fig). The presence of these sites suggests the possibility of auto-regulation of *Bdtra* splicing as observed in *C*. *capitata* [17].

A ClustalW multiple sequence alignment was performed based on the amino acid sequences of dipteran Tra. The alignment showed that Bdtra was more similar to Tra from other tephritid species than to *Drosophid* Tra (S1 Fig). Bdtra is 81.32% identical to *B*. *oleae* Tra, 54.15% identical to *C*. *capitata* Tra, 50.35% identical to *A*. *suspensa* Tra, but only 16.08% identical to *D*. *melanogaster* Tra. Indeed, *D*. *melanogaster* Tra is only 197 amino acids, much shorter than Bdtra. The Neighbour-joining tree of dipteran Tra showed that the species of Tephritidae, *Bactrocera* and *Ceratitis* clustered into one subgroup of the same branch, while *Anastrepha* clustered into a different subgroup of that same branch. The Tra proteins of tephritids are more closely related to *Musca* and *Lucilia* Tra than to *Drosophila* Tra (S5A Fig).

*Bdtra-2* was isolated with a similar method as for *Bdtra*. *Bdtra-2* was a transcript of 1332 bp in length (GenBank: KP342061) containing an open reading frame (ORF) of 756 bp, which encoded a putative protein of 251 amino acids with 96.02% identity to *B*. *oleae* Tra-2 and 90.08% identity to *C*. *capitata*Tra-2. Both males and females had only one transcript. *Bdtra-2* belongs to the Ser-Arg rich (SR) protein family and has a RNA recognition motif (RRM) flanked by two Arginine/Serine-rich domain (RS) as shown in S2 Fig. The RRM region and two ribonucleoprotein identifier sequences (RNPs) are highly conserved. BdTra-2 protein is similar to Tra-2 protein from other tephritids, such as *C*. *capitata*, *A*. *suspensa* and *B*. *oleae*, as well as to the one from other dipterans such as *M*. *domestica* and *L*. *cuprina*, but shows substantial divergence with Tra-2 from *D*. *melanogaster*. The Neighbour-joining tree of dipteran Tra-2 showed that the *Apis* and *Nasonia* clustered in a basal clade, *Drosophila* species in another clade and the other dipteran species in the third clade. Within the latter, *Musca* was in one branch and the other dipteran species in another. *Lucilia* clustered in one subgroup and the species of Tephritidae in another. Among the tephritids, *Ceratitis* and *Anastrepha* clustered into a subgroup of the same branch, *Bactrocera* clustered into a different subgroup of that same branch (S5B Fig).

Seven introns were detected when compared to the cDNA transcript with two overlapping fragments spanning the completed ORF of *Bdtra-2*. Further analyses of the *Bdtra-2* sequence indicate the presence of six putative intronic splicing silencer sequences (ISS) and thirteen putative RBP1 sites (Fig 1B; S4 Fig), which have been described as splicing activators or suppressors through binding with other RNA sequences [35]. These results suggest that the Bdtra-2 protein might be involved in female somatic sexual development through interaction with *Bdtra*.

### Expression patterns of *Bdtra* and *Bdtra-2*

**Fig 2:**
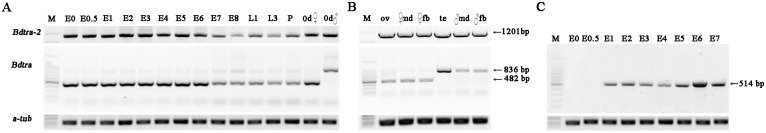
Expression patterns of *Bdtra* and *Bdtra-2*. (A, B) RT-PCR with *Bdtra* primers 225+/706- yielded male and female products with different sizes as indicated. RT-PCR with *Bdtra-2* primers 9+/1185- yielded male and female products of the same size. (C) RT-PCR with male-specific primer ms43+ and non-sex specific primer 706- to amplify the male-specific product. M, 100 bp DNA ladder (Takara); E0–E8, mixed sex embryos collected 0–0.5 h, 0.5–1 h, 1–2 h, 2–4 h, 4–6 h, 6–8 h, 8–10 h, 10–14 h, 14–21h and 24–48 h after egg laying, respectively; L1, first instar larvae of mixed sex; L3, third instar larvae of mixed sex; P, newly pupated pupae of mixed sex; 0d♀,newly emerged female adult; 0d♂, new emerged male adult; ♀md, midguts from 0d♀; ♂md, midguts from 0d♂; ♀fb, fat bodies from 0d♀; ♂fb, fat bodies from 0d♂; ov, ovaries; te, testes; *a-tub*, reference gene *a-tubulin*.

To analyze the expression patterns of *Bdtra*, RT-PCR was performed using primers 225+/706- to amplify different sized male and female forms of *Bdtra*. For *Bdtra-2*, primers 9+/1185- covered the whole ORF. RT-PCR was performed from mixed sex embryos, larvae, pupae and also adult females and males. A female product, a band of 482 bp, was readily detected, whereas a male product, a band of 836 bp was faint, although the embryos were presumably an equal mix of males and females (Fig 2A). Since the male form of *Bdtra* was difficult to detect when the female form was at significantly higher levels than the male form, the expression patterns were examined a second time using RT-PCR with male-specific primer ms43+ and the non-sex-specific primer 706- to amplify the male form of *Bdtra* in embryos. Male product was readily detected using male-specific primer ms43+ and the non-sex-specific primer 706-. A male product band of 514 bp was first detected in mixed sex embryos collected 1 to 2 h after the eggs were laid and in all later staged embryos (Fig 2C). In addition, only the female form of *Bdtra* was detected in female adults and their tissues, such as ovaries, midguts and fat bodies. The male form of *Bdtra*was also only detected in male adults and their tissues, such as testes, midguts and fat bodies (Fig 2B). For *Bdtra-2*, a single band of the expected size (1201 bp) was amplified in all samples (Fig 2A and 2B).

### RNAi knock-down of *Bdtra* and *Bdtra-2*

**Fig 3:**
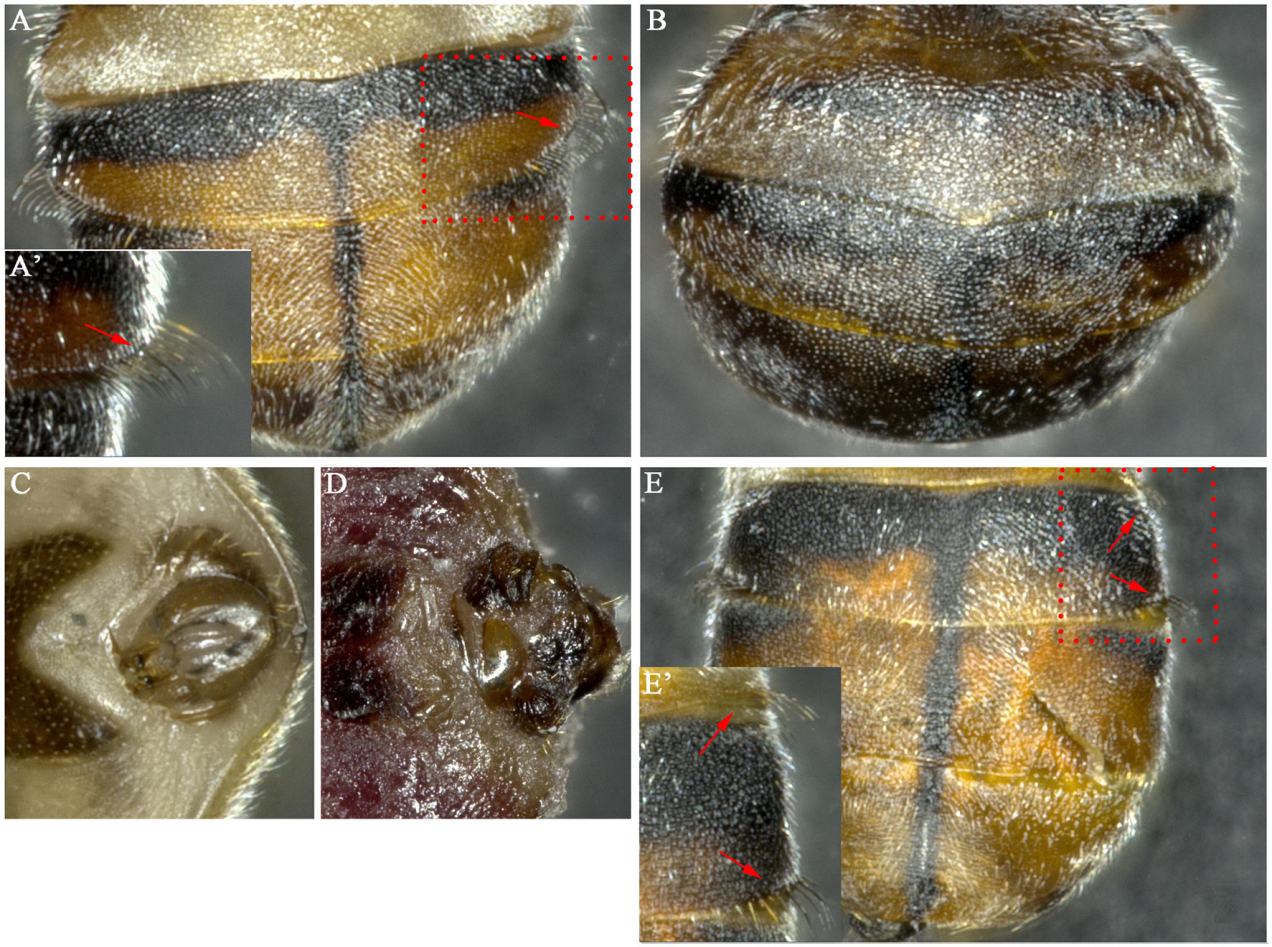
Phenotypes analyses of RNAi intersex individuals. (A) Wild-type male with 25 black short bristles on each lower side of the third tergum, magnification of the red dotted section in A is shown in A’; (B) Intersex individual with male genitalia and female dorsal phenotype without bristles on both sides; (C) Normal male genitalia in XY males; (D) Malformed male genitalia of one intersex individual; (E) One XY male with an excessive row of bristles on the right lower side of the second tergum, magnification of the red dotted section in E was shown in E’. A, B, E: dorsal view; C-D: ventral view; arrows indicate the position of bristles.

To characterize the function of *Bdtra* and *Bdtra-2* in sex determination, RNAi was employed to knock-down these two genes. Three dsRNAs (ds*Bdtra*_79, ds*Bdtra*_613 and ds*Bdtra-2*_422) were injected into the posterior end of newly laid eggs. In *B*. *dorsalis* adults, males can be differentiated from females by external genitalia and a row of 22–28 bristles on each lower side of the third tergum (Fig 3A). In contrast, intersex individuals are sexual mosaics, with male external genitalia and a female dorsal view without bristle (Fig 3B). As shown in Table 1, besides a few of intersex individuals, more than 90% males developed from embryos injected with ds*Bdtra*_79, ds*Bdtra*_613 and ds*Bdtra-2*_422, respectively. Since the sex ratio of adults that developed from uninjected and ds*EGFP* injected embryos was nearly 1.0 (S1 File), our results suggest that knock-down of *Bdtra* or *Bdtra-2* during early development induced masculinization efficiently. The intersex phenotype was probably due to partial transformation of the injected female embryos from incomplete knock-down of *tra* or *tra-2*. Notably, one XY male derived from the ds*Bdtra*_79 injection showed an excessive row of bristles on the lower right side of the second tergum (Fig 3E). The XX-reversed males and intersex individuals had male genitalia similar to the XY males (Fig 3C) except for one intersex individual from the ds*Bdtra*_ 613 injection (Fig 3D). These results demonstrate that both *Bdtra* and *Bdtra-2* are indispensable for female somatic sexual development in *B*. *dorsalis*.

**Table 1:** Statistics on knock-down phenotypes of *Bdtra* and *Bdtra-2* by dsRNA injection.

Males from	Injected eggs	Adults obtained	Females ^a^ (%)	Males ^a^ (%)	Intersex ^a^ (%)
uninjected	400	328	172(52.4)	156(47.6)	0
ds*EGFP*	400	78	40(51.3)	38(48.7)	0
ds*Bdtra* 79	478	61	2(3.3)	55(90.2)	4(6.6)
ds*Bdtra* 613	461	72	0	69(95.8)	3(4.2)
ds*Bdtra-2*_422	500	61	2(3.3)	56(91.8)	3(4.9)

^a^The percentage of the phenotypes is divided into the number of adults obtained from each treatment.

### Fertility test of RNAi males

To investigate the fertility, RNAi-generated males were backcrossed to virgin wild type females in separate cages for each dsRNA treatment. As shown in Table 2, for uninjected and ds*EGFP*-injected groups, 100% and 94.3% of the cages contained bisexual progeny at a ratio of nearly 1.0 (S1 File). While in the cages that contained males which developed from embryos injected with ds*Bdtra*_79, ds*Bdtra*_613, ds*Bdtra-2*_422, nearly 80% of the RNAi males mated and the mated females laid eggs, but only 42.9%, 48.6 and 40% of the RNAi males could produce progeny in each treatment (Table 2). Among the RNAi males with progeny, a little more than 20% of the RNAi males could only produce female progeny (Table 2). These males were considered to be XX-reversed males. In addition, all of the intersex individuals could not produce progeny due to a lack of females laying eggs or eggs failing to hatch.

**Table 2:** Backcrosses of XX and XY males from dsRNA injected eggs were backcrossed to wild females in 35 cages.

Treatment	Mating (%)	Spawning (%)	Hatching (%)	Adults emerging	Female-only emerging ^a^ (%)
Uninjected (35)	35(100)	35(100)	35(100)	35(100)	0
ds*EGFP* (35)	34(97.1)	34(97.1)	33(94.3)	33(94.3)	0
ds*Bdtra*79 (35)	29(82.9)	29(82.9)	15(42.9)	15(42.9)	3(20)
ds*Bdtra*613 (35)	30(85.7)	28(80)	17(48.6)	17(48.6)	4(23.5)
ds*Bdtra-2*_422 (35)	28(80)	26(74.3)	14(40)	14(40)	3(21.4)

^a^The percentage of Female-only emerging is calculated by dividing into the number of cages with adults emerging.

**Fig 4:**
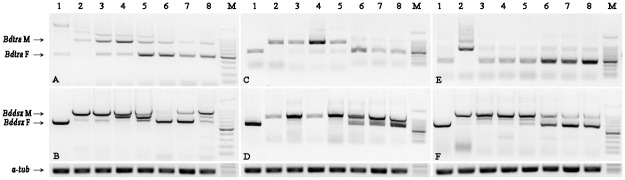
Expression patterns of *Bdtra* and *Bddsx* in RNAi males. (A, B) Injection with ds*Bdtra*_79. (C, D) Injection with ds*Bdtra*_613. (E, F) Injection with ds*Bdtra-2*_422. (A, C, E) Detection of the transcripts of *Bdtra*. (B, D, F) Detection of the transcripts of *Bddsx*. Abbreviations and notes: *Bdtra* M and *Bddsx* M: male-specific splicing forms of *Bdtra* and *Bddsx* respectively; *Bdtra* F and *Bddsx* F: female-specific splicing forms of *Bdtra* and *Bddsx* respectively; *a-tub*, reference gene a-*tubulin*. The band between *Bddsx* M and *Bddsx* F was a male transcript with the size of 130bp shorter than *Bddsx* M in 3’UTR. Lane 1: female fly from ds*EGFP*-injection; Lane2: male fly from ds*EGFP*-injection; Lane 3, 4, 5: XX-reversed males; Lane 6, 7, 8; intersex individuals; Lane M: Takara 100bp DNA ladder.

In order to investigate the effects of *Bdtra-2* and *Bdtra* on *Bdtra* and their downstream target gene *Bddsx*, the transcript levels of *Bdtra* and *Bddsx* were investigated in XX-reversed males and intersex individuals. As shown in Fig 4, both male and female forms of *Bdtra* were detected in both XX-reversed males and intersex individuals (Fig 4A, 4C and 4E). The bands of female-specific *Bdtra* in intersex individuals were stronger than that of male-specific *Bdtra*. For *Bddsx*, male-specific *Bddsx* transcripts were detected, whereas female-specific *Bddsx* transcripts were undetected or produced a very faint band in the XX-reversed males. In intersex individuals, both male and female forms of *Bddsx* were detected, however, the bands of female-specific *Bddsx* were much stronger than that in the XX-reversed males (Fig 4B, 4D and 4F).

**Fig 5:**
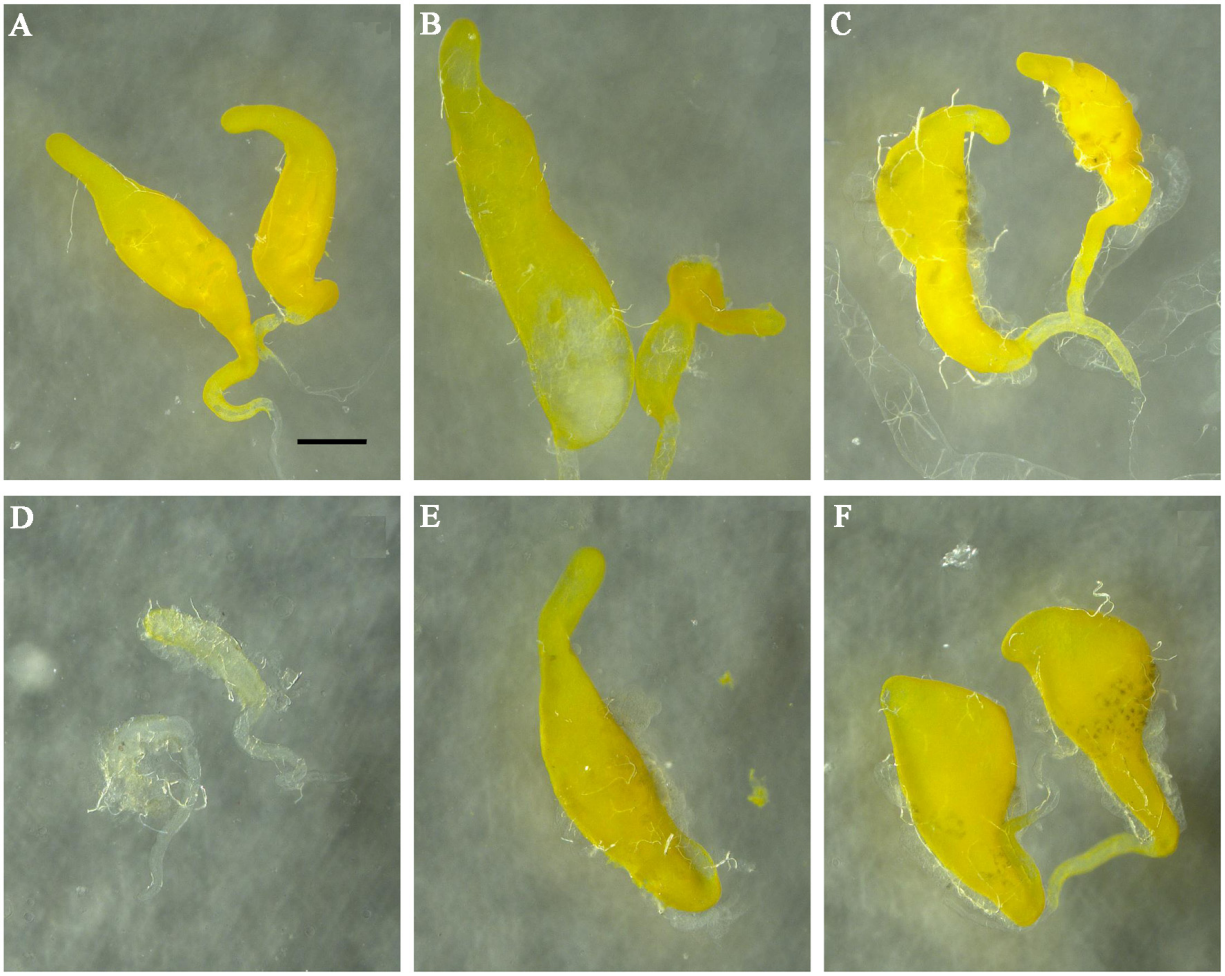
Internal genital structures of RNAi males. Testes of XY males (A) and testes that demonstrated various types of abnormal morphology such as a well-developed testis plus a poorly developed testis (B), hypertrophic testes (C, F), underdeveloped testes (D), single testis (E) from XX-reversed males and intersex individuals. Similar results were obtained for ds*Bdtra* and ds*Bdtra-2* injections. Scale bars are all the same and indicate 0.2mm.

All RNAi males involved in the fertility test were dissected to examine the internal genital structures. XX-reversed males and intersex individuals had no ovaries, but testes of a similar structure were observed. In contrast to testes of XY males (Fig 5A), XX-reversed males and intersex individuals showed aberrant gonads, such as a well-developed testis plus a poorly developed testis (Fig 5B), hypertrophic testes (Fig 5C and 5F), underdeveloped testes (Fig 5D) or only one testis (Fig 5E). After dissecting the testes, motile sperm were observed in all cases.

## Discussion

In this work, the isolation, expression and function of *B*. *dorsalis tra* and *tra-2* genes were reported. The genomic structure of *Bdtra* and *Bdtra-2* are very similar to that of *C*. *capitata* [17–18] and *A*.*suspensa* [25]. *Bdtra* has sex-specific transcripts: one female-specific transcript, which encodes a functional Tra protein of 422 amino acids in length, and two male-specific transcripts with in-frame stop codons, which leads to the production of non-functional, truncated Tra protein. In contrast, *Bdtra-2* has a non-sex-specific transcript which leads to the production of Tra-2 protein that is 251 amino acids in length. The BdTra-2 protein shows classic features of RNA-binding proteins, with a conserved RRM domain and the presence of ISS and RBP1 sites. Tra protein seems to lack an RNA binding domain, and its effects on splicing regulation require interactions with other proteins containing RNA-binding domains, such as Tra-2 [36]. In the tephritids, housefly and calliphorids *tra* genes, at least one Tra/Tra-2 binding site was found in the male-specific exon that is downstream of the first protein coding exon [17, 20, 37]. This suggests that binding of Tra/Tra-2 to the male-specific exon plays an important role in splice site selection of *tra* in females. In *Bdtra*, two Tra/Tra-2 binding sites were found in the first male-specific exon. Tra/Tra-2 binding sites were also found in *Bddsx* [32]. These results suggest that the Tra/Tra-2 complex in *B*. *dorsalis*, similar to that in *D*. *melanogaster* and *C*. *capitata*, regulates the alternative splicing of *tra* and *dsx* by specifically interacting with their pre-mRNAs.

In medfly, housefly and *L*. *cuprina*, female forms of *tra* are maternally transcribed for the presence of female forms of *tra* in very early precellular embryos of mixed sex, whereas male form of *tra* starts the transcription at different developmental stage in different species, such as embryos of 4 and 5 h after egg laying in the medfly and the first instar larvae stage in *L*. *cuprina* [17, 20, 22, 38]. In addition, in medfly, male and female *tra* transcripts were found in both male and female embryos between 4 to 8 h after the eggs were laid; by 9 h after the eggs were laid, only the sex-specific transcripts were found, which suggested that the postulated *M*-factor acts by inhibiting the Tra protein activity [38]. In the oriental fruit fly, the female form of *Bdtra* was detected in newly emerged adult ovaries and embryos of 0–0.5 h after egg laying, which suggested that the female form of *Bdtra* was maternally inherited in the embryos as the flies above. The male *tra* transcript of the oriental fruit fly was detected from 1–2 h embryos onward, much earlier than that of the medfly and *L*. *cuprina*.

In contrast to *Bdtra*, *Bdtra-2* produces a non-sex-specific splicing pattern, a single band was detected in both sexes at all the developmental stages and in different adult tissues analyzed. It is the same as that in *M*. *domestica* [19], *C*. *capitata* [18] and *Anastrepha* fruit flies [24], but differs in *D*. *melanogaster* as two *tra-2* transcripts are detected in male and female, two other *tra-2* transcripts are present only in male germ cells, due to alternative splicing and transcription start sites [39–40]. Among these transcripts, one of them is transcribed at a high level in the germ line and is indispensable to male fertility [41]. Thus, the function of *tra-2* on male fertility might be lost in *B*. *dorsalis*.

Further characterization of *Bdtra* and *Bdtra-2* was performed by transient knock-down their transcripts using dsRNA. Nearly 500 eggs were injected in each treatment. Knock-down of *Bdtra* or *Bdtra-2* by the injection of ds*Bdtra*_79, ds*Bdtra*_613 or ds*Bdtra-2*_422 effectively led to sex reversion of XX females to phenotypic males. In particular, the injection of ds*Bdtra*_613 resulted in 95.8% males (XY and XX males) and three intersex individuals (4.2%). Moreover, male-specific transcripts of *Bdtra* and *Bddsx* were detected in XX-reversed males and intersex individuals. Upon dissection, both XX reversed males and intersex individuals have no clear female gonadal morphology but the testes similar structure. The fertility test of RNAi males including the intersex individuals showed that the XX-reversed males could mate and produce only female progeny, whereas no progeny was produced from the intersex individuals in spite of the presence of motile sperm. These results suggest that the level of masculinization might affect fertility. In addition, eggs from almost half of the cages failed to hatch. This may be attributed to incomplete knock-down of *Bdtra* and *Bdtra-2* for the infertile mating of XX-reversed males or their roles in other embryonic developmental events in *B*. *dorsalis* as the similar results also occurred when knock-down of *tra-2* during the early embryonic stage of *A*. *mellifera* resulted in the death of embryos after approximately 70 h of development [42] and parental RNAi of *tra-2* in *Tribolium castaneum* led to hatching failure of a few eggs [43]. The mechanism of the function of *tra* and *tra-2* in embryonic development is unknown at this time. However, in *T*. *castaneum*, the role of *tra-2* during embryonic development was considered to be related to regulation of Tra/Tra-2 target genes coding for proteins such as those involved in the formation of the dosage compensation complex [43].

The isolation of *Bdtra* will facilitate the development of a transgenic sexing system. In *C*. *capitata*, a differential splicing intron of *tra* was introduced in a construct to transfer female-specific splicing to a bacterial toxin gene, leading to the development of a female-specific lethal strain [26]. In addition, tetracycline-suppressible transgenic embryonic sexing systems were developed for *A*. *suspensa* [27] and *C*. *capitata* [28]. In this study, the sex reversion induced by either *Bdtra* or *Bdtra-2* in *B*. *dorsalis* was highly efficient. These XX-reversed males could mate with wild type females, which is a prerequisite for competition in future SIT programs. These findings suggest that it may be possible and promising to develop heritable *Bdtra* or *Bdtra-2* deficient strains through a transgene-based RNAi strategy for the production of male-only strains. The successfully tested male-only strain could then be irradiated to render all progeny sterile. Alternatively, fertile male flies carrying a conditional female transformation system could be used for a RIDL program. Indeed, modeling [44] suggests such a strategy could be very efficient system for population suppression, which would be advantageous when dealing with a species such as *B*. *dorsalis* that can be present in high densities in fruit-growing regions.

## Supporting Information

S1 Fig Alignment of Tra proteins of insect species from Diptera. Identical and similar amino acids are shown in black and gray shade. Arrows indicate the position of primers to amplify the cDNA fragment of *Bdtra*.
(TIF)

S2 Fig Alignment of Tra-2 proteins of insect species from Diptera. The RNA recognition motif (RRM) is in black shade, flanked by the N- and C-terminal arginine/serine (RS)-rich regions in grey shade. Two ribonucleoprotein identifier sequences (RNPs) are shown in the box with red dotted lines. Arrows show the position of primers to amplify the cDNA fragment of *Bdtra-2*.
(TIF)

S3 Fig Detailed structure of the differentially spliced intron of *Bdtra*. Three male-specific exons are shown in red, green and yellow colored letters. Eight putative Tra/Tra-2 binding sites (red underlined), five putative intronic splicing silencer (ISS) sites (blue underlined) and four putative RBP1 sites (in box) were found.
(TIF)

S4 Fig Detailed structure of *Bdtra-2*. Six putative intronic splicing silencer (ISS) sequences (underlined) and thirteen putative RBP1 sites (in box) were identified. Exons are shown in black colored letters; translation start and stop codons are depicted in red. Introns are shown in blue and italic.
(TIF)

S5 Fig Neighbour-joining trees of dipteran Tra (A) and Tra-2 (B) amino acid sequences. The numbers next to the branches represent bootstrap support value from 1000 replicates. The scale represents the mean character distance. The topology was rooted with the Tra or Tra2 protein from the hymenopterans *A*. *mellifera* and *N*. *vitripennis*.
(TIF)

S1 Table Primers. (DOCX)

S1 File The numbers of male and female progeny of RNAi males backcrossing to wide-type females. (XLSX)
