# Supplementary figures and images for: RNAi-Mediated Knock-Down of transformer and transformer 2 to Generate Male-Only Progeny in the Oriental Fruit Fly, Bactrocera dorsalis (Hendel)
Source: PLoS One. 2015 Jun 9;10(6):e0128892. doi: 10.1371/journal.pone.0128892 (PMC4461288; doi:10.1371/journal.pone.0128892)

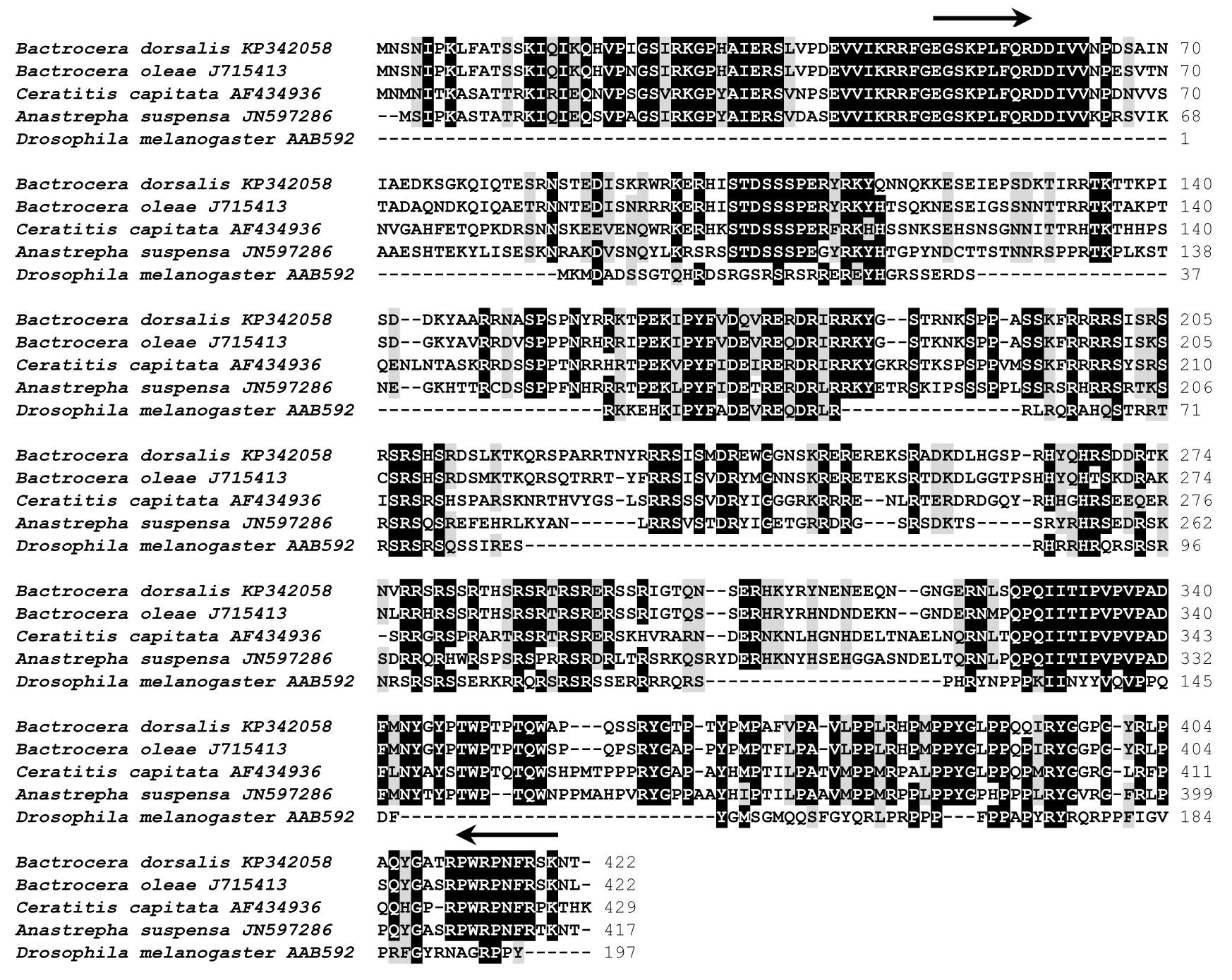

Supplement: S1 Fig — Identical and similar amino acids are shown in black and gray shade. Arrows indicate the position of primers to amplify the cDNA fragment of Bdtra. (TIF) [file pone.0128892.s001.tif]

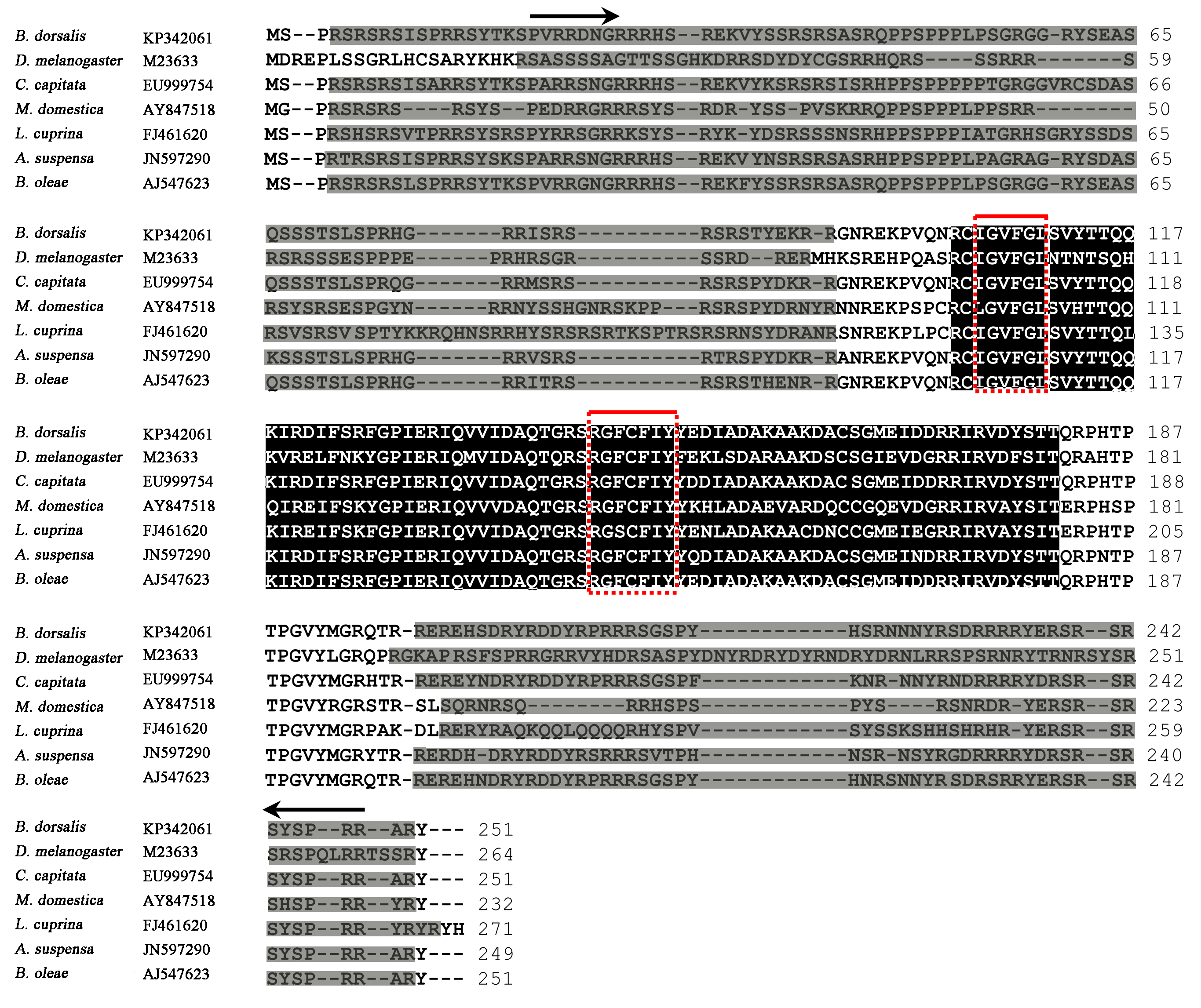

Supplement: S2 Fig — The RNA recognition motif (RRM) is in black shade, flanked by the N- and C-terminal arginine/serine (RS)-rich regions in grey shade. Two ribonucleoprotein identifier sequences (RNPs) are shown in the box with red dotted lines. Arrows show the position of primers to amplify the cDNA fragment of Bdtra-2. (TIF) [file pone.0128892.s002.tif]

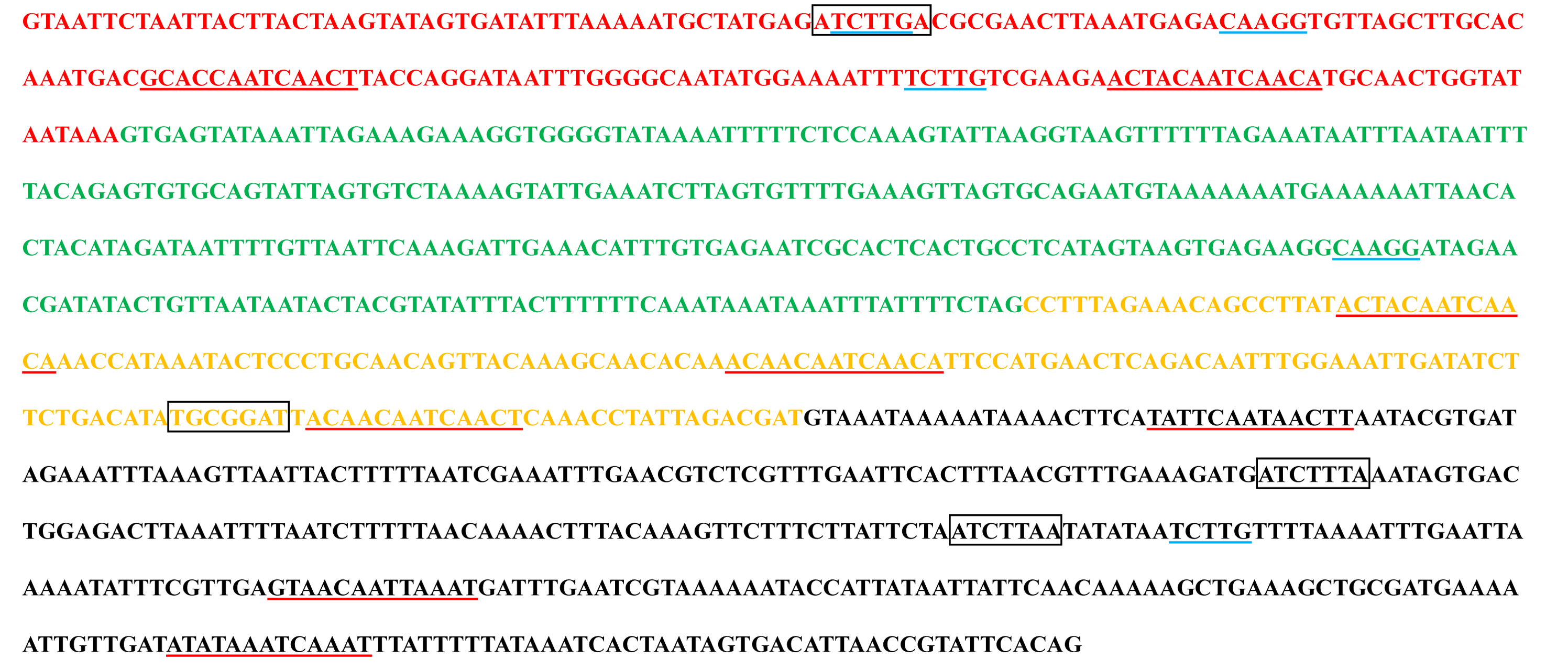

Supplement: S3 Fig — Three male-specific exons are shown in red, green and yellow colored letters. Eight putative Tra/Tra-2 binding sites (red underlined), five putative intronic splicing silencer (ISS) sites (blue underlined) and four putative RBP1 sites (in box) were found. (TIF) [file pone.0128892.s003.tif]

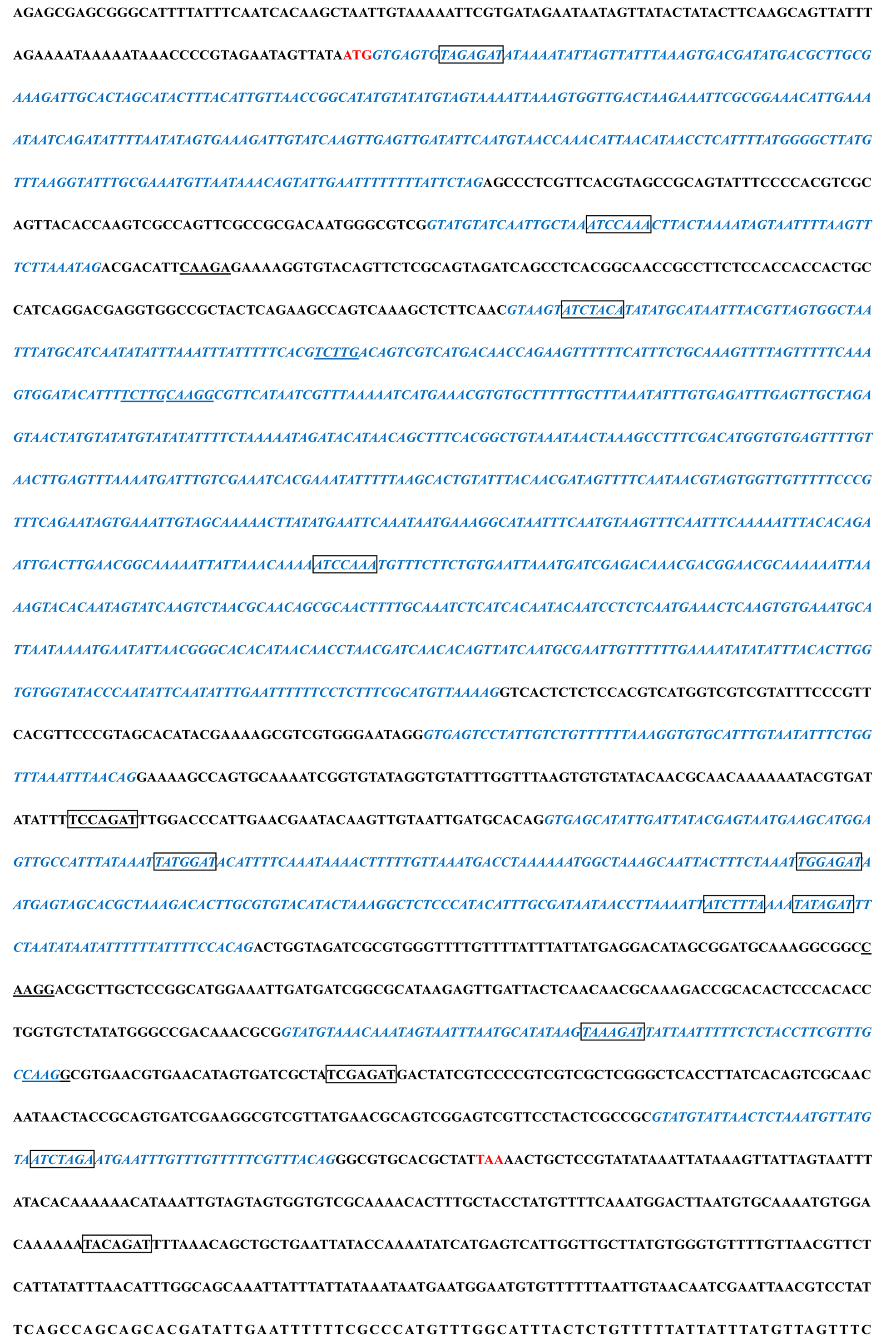

Supplement: S4 Fig — Six putative intronic splicing silencer (ISS) sequences (underlined) and thirteen putative RBP1 sites (in box) were identified. Exons are shown in black colored letters; translation start and stop codons are depicted in red. Introns are shown in blue and italic. (TIF) [file pone.0128892.s004.tif]

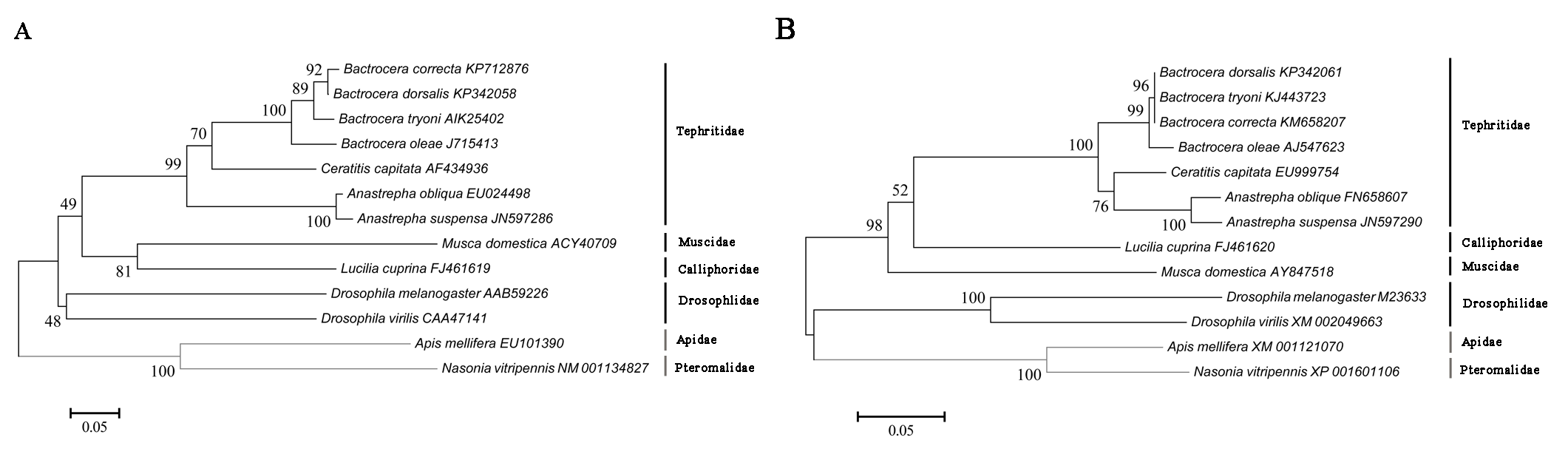

Supplement: S5 Fig — The numbers next to the branches represent bootstrap support value from 1000 replicates. The scale represents the mean character distance. The topology was rooted with the Tra or Tra2 protein from the hymenopterans A. mellifera and N. vitripennis. (TIF) [file pone.0128892.s005.tif]
