# Supplementary material for: RNAi-Mediated Knock-Down of transformer and transformer 2 to Generate Male-Only Progeny in the Oriental Fruit Fly, Bactrocera dorsalis (Hendel)
Source: PLoS One. 2015 Jun 9;10(6):e0128892. doi: 10.1371/journal.pone.0128892 (PMC4461288; doi:10.1371/journal.pone.0128892)
Supplement: S1 Table — (DOCX) [file pone.0128892.s006.docx]

**Table S1. Primers**

| Name | Sequences are shown in 5’ to 3’ |
| --- | --- |
| 260+ | GGTTCAAAGCCTTTGTTTCA |
| 1329- | TTGATCGAAAATTGGGTCGC |
| 286+ | GCAGCTCGACGTAACGCATCACCA |
| 1043+ | CGTTACGGCGGACCTGGATATAGATTAC |
| 514- | GCGACGATAATTAGTGCGACGAGCTGG |
| 286- | TGGTGATGCGTTACGTCGAGCTGC |
| 3+ | GGGGATCGCGACAAACAGCAT |
| 225+ | GCAACATGTGCCCATTGGCTCA |
| 655+ | GTAGACCAAGTAAGGGAGCGAGACAGAAT |
| Ms43+ | CTCCCTGCAACAGTTACAAAGCAACAC |
| 706- | GAATTCTGTCTCGCTCCCTTACTTGGTCT |
| 299- | ACAACTTCGTCTGGAACTAAACTGCGT |
| 1285- | AGGCAGGCATAGGATAAGTGGGAGT |
| AF2+ | AGGCAGGCATAGGATAAGTGGGAGT |
| AR1- | GCCGCGGCGAATAGGAACGA |
| 228- | GCGTCGTGGGAATAGGGAAAAGCC |
| 354+ | GATCACTATGTTCACGTTCACGCCGCG |
| 9+ | AGAGCGAGCGGGCATTTTATTTC |
| 366- | CGGGAACGTGAACGGGAAATAC |
| 358+ | TGGTCGTCGTATTTCCCGTTCAC |
| 1185- | ATCGTGCTGCTGGCTGAATAGGAC |
| 1074+ | GCAGGACGAGCAAAGGGTTCT |
| f1072- | CAGATTGTGTTGACGGGAGTATTCG |
| m2015- | CGTTGCGACTGTTGTTGCTGTTAC |
| 79+ | TAATACGACTCACTATAGGGAGGAAGGGTCCTCACGCTAT |
| 576- | TAATACGACTCACTATAGGGTGCTGGAGGCGATTTATTTC |
| 613+ | TAATACGACTCACTATAGGGTCACGCTCACGATCTCACTC |
| 1205- | TAATACGACTCACTATAGGGCTATATCCAGGTCCGCCGTA |
| 422+ | TAATACGACTCACTATAGGGGTAGATCGCGTGGGTTTTGT |
| 732- | TAATACGACTCACTATAGGGGTAGGAACGACTCCGACTGC |
| 27+ | TAATACGACTCACTATAGGGGTGAGCAAGGGCGAGGAG |
| 688- | TAATACGACTCACTATAGGGCGGCGGTCACGAACTCCAG |
| *α-tub*+ | CGCATTCATGGTTGATAACG |
| *α-tub*- | GGGCACCAAGTTAGTCTGGA |
